# Prehospital transfusion training in Canada: a national survey of critical care transport organizations

**DOI:** 10.1186/s13049-025-01435-x

**Published:** 2025-07-01

**Authors:** Pierre-Marc Dion, Andy Pan, Andrew Beckett, Kanwal Singh, Adam Greene, Axel Benhamed, Melissa McGowan, Brodie Nolan

**Affiliations:** 1 Faculty of Medicine, University of Ottawa, Ottawa, ON Canada; 2 Institut du Savoir Montfort, Ottawa, ON Canada; 3 Hôpital Montfort, Ottawa, ON Canada; 4 Department of Emergency Medicine, The Ottawa Hospital, Ottawa, ON Canada; 5 Ornge Air Ambulance and Critical Care Transport, Mississauga, ON Canada; 6 Department of Surgery, St. Michael’s Hospital, Toronto, ON Canada; 7 FIRST60, Li Ka Shing Knowledge Institute, St. Michael’s Hospital, Unity Health Toronto, Toronto, ON Canada; 8 Defence Research and Development Canada, Toronto, ON Canada; 9 British Columbia Emergency Health Services, Vancouver, BC Canada; 10 School of Medicine, Cardiff University, Cardiff, Wales, UK; 11 Département d’urgence, Hôpital de l’Enfant-Jésus, CHU de Québec - Université Laval, Québec, Canada; 12 Service d’évacuations aéromédicales du Québec (EVAQ), CHU de Québec- Université Laval, Québec, Québec Canada; 13 Department of Emergency Medicine, St. Michael’s Hospital, Toronto, ON Canada; 14 Division of Emergency Medicine, Department of Medicine, University of Toronto, Toronto, ON Canada

**Keywords:** Blood transfusion, Air ambulances, Emergency medical services, Critical care, Simulation training, Medical education

## Abstract

**Background:**

Hemorrhagic shock is a leading cause of preventable death, and prehospital transfusion has been associated with improved outcomes in select trauma and medical patients. In Canada, several Critical Care Transport Organizations (CCTOs) have implemented prehospital transfusion programs to reduce geographic disparities in access to definitive care. However, limited evidence exists on how providers are trained to deliver this intervention. While simulation-based education and instructional design features improve skill retention in other contexts, their application in prehospital transfusion training has not been systematically evaluated. This study aimed to assess current training practices among Canadian CCTOs and evaluate their effectiveness.

**Methods:**

We conducted a cross-sectional survey across all Canadian CCTOs. Data were analyzed descriptively using the Kirkpatrick Model framework, which evaluates training effectiveness across four levels: learner satisfaction, knowledge acquisition, behaviour change, and patient outcomes. Reporting followed the Consensus-based checklist for reporting of survey studies (CROSS) guidelines.

**Results:**

All seven Canadian CCTOs with active prehospital transfusion programs participated (100% response rate), with respondents including one transport physician, three registered nurses, and three critical care paramedics per organization. Programs represented fixed-wing, rotor-wing, and land-based transport systems operating in urban, suburban, rural, and remote settings. Training approaches varied across CCTOs. Checklists were universally used to assess competency, with four organizations incorporating additional tools such as global rating scales and scenario-based evaluations. Recertification practices were inconsistent: one CCTO required annual recertification, three used bi-annual reviews, and three had no formal recertification process. Using the Kirkpatrick Model, all seven CCTOs demonstrated Level 1 (Reaction) through provision of training; five used structured feedback mechanisms, while two relied on informal feedback. At Level 2 (Learning), six organizations used didactics, practical workshops, and field training, while one relied solely on mentorship. Level 3 (Behaviour) evaluations were conducted by four CCTOs, primarily through structured assessments; three relied on documentation audits or informal peer review. No CCTOs reported Level 4 (Results) assessments through tracking of patient outcomes related to transfusion.

**Conclusions:**

Considerable variability exists in prehospital transfusion training across Canadian CCTOs. Establishing training standards may support improved provider preparedness and contribute to enhanced patient care, although further evaluation is needed.

**Supplementary Information:**

The online version contains supplementary material available at 10.1186/s13049-025-01435-x.

## Background

Hemorrhagic shock is a leading cause of preventable death globally, and early access to blood products has been increasingly recognized as a beneficial intervention for selected trauma and medical patients [1–3]. In geographically large and sparsely populated countries such as Canada, access to definitive care is often delayed due to regional disparities in healthcare infrastructure [4,5]. Specialized care is typically concentrated in secondary-, tertiary-, and quaternary-level hospitals located in urban centers, resulting in prolonged transport times for patients in rural and remote areas. To address this challenge, several Critical Care Transport Organizations (CCTOs) have implemented prehospital transfusion programs to reduce geographic disparities whilst accessing definitive care [6–8]. Although early transfusion has been associated with improved outcomes, the evidence specific to prehospital settings remains limited and inconclusive [9].

The effectiveness of prehospital blood transfusion depends on the clinical competence of healthcare providers, including their ability to recognize indications, perform procedures, and manage transfusions safely in dynamic environments [10–12]. Structured resuscitation training programs are designed to support these competencies; however, studies have shown that procedural skills often deteriorate within months if not regularly reinforced. Educational interventions such as simulation-based education, spaced repetition, and mastery learning have been shown to improve long-term skill retention in in-hospital settings, although their specific application to prehospital transfusion training remains underexplored [13]. These approaches may be relevant for prehospital training programs aimed at supporting skill retention and readiness in high-acuity settings.

Despite this, standardized training programs tailored specifically to prehospital transfusion remain limited. The current body of literature consists mainly of observational studies and case series, offering little consensus on optimal training methods [14–19]. This deficiency highlights the need for a structured framework to assess and enhance the training methodologies for prehospital care providers. This gap highlights the need for a structured framework to guide evaluation and development of transfusion training initiatives. The Kirkpatrick Model is a widely used framework for evaluating training effectiveness across four domains: learner satisfaction, knowledge acquisition, behavior change, and results [20].

In addition, educational frameworks that incorporate cognitive task analysis, simulation, and other evidence-informed strategies have shown promise in improving performance in complex clinical environments [21,22]. However, the extent to which these approaches have been adopted within Canadian CCTOs is unclear.

## Methods

This cross-sectional survey-based study aimed to describe the current state of prehospital transfusion training among Canadian CCTOs and to evaluate these programs using the Kirkpatrick Model. A secondary objective was to examine the integration of instructional design features that may influence training effectiveness. The findings aim to inform future efforts in curriculum development and contribute to the establishment of best practices in prehospital transfusion education. Data was collected between February 16 and November 30, 2024. We reported our findings following the *Consensus-based checklist for reporting of survey studies* (CROSS) (Appendix 1) [23].

### Study setting

Critical care transport in Canada falls under provincial jurisdiction, which creates variability in governance structures, team composition, and training requirements [6–8]. Each province manages critical care transport differently by employing multidisciplinary teams composed of transport physicians, registered nurses, and specialized paramedics in various configurations [6,7]. These teams deliver prehospital care and transfer critically ill patients between facilities across diverse environments, including a mix of rotor-wing (helicopter), fixed-wing (airplane), and land-based (ground ambulance) resources. Crew compositions within Canadian CCTOs commonly include paramedic-only teams, combined registered nurse and paramedic teams, and physician-registered nurse teams, reflecting regional differences in resources and clinical requirements [6,7].

Given these regional differences, standardization of prehospital transfusion practices and protocols is not universally implemented across Canada [6,24]. To address these challenges, the Canadian Prehospital and Transport Transfusion (CAN-PATT) network was established to facilitate collaboration, protocol development, and training optimization among prehospital and Critical Care Transport Organizations across Canada [6]. This network serves as a coordinating body for research, education, and best practices in prehospital transfusion, involving clinicians and researchers from across the country [6].

## Survey design

A multidisciplinary team with expertise in critical care transport, trauma, and transfusion medicine developed a preliminary survey to ensure clarity, relevance, and comprehensiveness. Pilot testing and iterative feedback informed refinements in question phrasing, removal of redundancies, and the inclusion of key elements to optimize the evaluation of transfusion training programs across CCTOs. The final survey included 31 items addressing training delivery methods, simulation use, instructional design features, competency maintenance, and recertification practices (Appendix 2). Participants were initially identified through the CAN-PATT network, and snowball sampling was employed to reach additional qualified respondents. The survey was administered anonymously via a secure online platform (Jotform^®^, San Francisco, CA, USA), and data were subsequently downloaded to a secure institutional server for analysis (Microsoft 365 SharePoint, Microsoft Corporation Inc., Redmond, Washington).

## Participants and recruitment

We surveyed prehospital personnel involved in the development, implementation, and delivery of transfusion training within Canadian CCTOs. Eligible participants included clinicians, educators, and leaders responsible for clinical oversight, curriculum development, competency assessment, and simulation-based education. To ensure a comprehensive understanding of training practices, priority was given to respondents directly engaged in the instructional design and evaluation of transfusion training programs.

Given the specific nature of the study and the use of convenience and snowball sampling methods, a formal sample size calculation was not required. However, based on previous survey response trends in healthcare research, a 35% response rate was anticipated among invited Canadian CCTOs [25]. Given the focus on key educators and training personnel, a minimum of one representative per CCTO was expected to ensure adequate coverage of transfusion training practices across the national network.

## Outcome measures

The primary outcome of this study was to describe prehospital transfusion training practices across Canadian Critical Care Transport Organizations (CCTOs), with a focus on program structure, instructional design features, and competency assessment methods. Specific areas of interest included the educational modalities used, the integration of simulation and technology-enhanced learning, the inclusion of instructional design strategies, the use of simulated blood products, and approaches to competency maintenance, including procedural volume requirements and recertification intervals.

To evaluate training content and delivery, the survey assessed the types of educational interventions employed by CCTOs, including online modules, in-class lectures, practical workshops, simulation-based sessions, and field mentorship. Given the strong evidence supporting simulation as an effective method for enhancing procedural competency and decision-making, we examined the extent and type of simulation integrated into prehospital transfusion training programs. This included the use of part-task trainers, screen-based modules, high-fidelity mannequins, and emerging technologies such as augmented, virtual, and mixed reality [13,26–28].

Instructional design features were also examined to determine whether CCTOs applied evidence-based strategies known to enhance skill acquisition and retention [21]. These included cognitive task analysis, mastery learning, distributed practice, expert instructor presence, exposure to clinical variation, group versus independent learning, individualized pacing, and multimodal teaching strategies. The inclusion of repetitive practice, variable task difficulty, and structured feedback mechanisms was also explored [21].

Competency assessment and maintenance practices were evaluated by identifying the tools used to measure learning outcomes and provider performance. These included time-based assessments, multiple-choice knowledge tests, psychomotor assessments (e.g., hand-motion and visuospatial analysis), checklists, global rating scales, success rate tracking, and participant feedback [29,30]. We also reviewed each CCTO’s policies on minimum procedural thresholds for transfusion, intravenous (IV) and intraosseous (IO) access, as well as training renewal or recertification cycles.

While this study did not assess the specific types of blood products administered, the frequency of transfusion, or the clinical indications guiding their use, these elements have been examined in recent national studies [6–8]. These findings provide critical context for understanding the operational environments in which transfusion training occurs in Canadian CCTOs.

Finally, although direct patient outcomes were not measured, we explored whether organizations had systems in place to monitor transfusion-related performance. This included tracking protocol adherence, procedural success rates, and transfusion-related adverse events. To structure our evaluation of training effectiveness, we applied the Kirkpatrick Model framework, which categorizes outcomes into four levels: learner satisfaction (Level 1), knowledge and skill acquisition (Level 2), behavior change in clinical settings (Level 3), and organizational or patient-level results (Level 4) [20].

### Data analysis

We exported all survey responses to Microsoft Excel 365 (Version 16.95, Microsoft Corporation Inc., Redmond, Washington) for cleaning and analysis. We performed descriptive statistics to summarize categorical and continuous variables. We calculated frequencies and proportions to summarize the data. We did not collect any continuous variables. There were no missing responses, so we did not perform any data imputation. Given the descriptive aim and small sample size of the study, we did not conduct inferential statistical tests. We organized the results using the Kirkpatrick Model framework to align training practices with levels of evaluation.

### Ethical approval

This study received ethics approval from the Unity Health Toronto Research Ethics Board (REB #23–270).

## Results

We identified and contacted seven CCTOs across Canada with prehospital blood transfusion programs, all of which participated in the survey, resulting in a 100% response rate. There was no missing data. The following seven CCTOs with active prehospital transfusion programs participated in this study: British Columbia Emergency Health Services (BCEHS), Shock Trauma Air Rescue Service (STARS) Alberta, STARS Saskatchewan, STARS Manitoba, Ornge (Ontario), Évacuations aéromédicales du Québec (EVAQ), and Emergency Health Services LifeFlight (Nova Scotia). Respondents included one transport physician (14%), three registered nurses (43%), and three critical care paramedics (43%), all of whom were involved in the development and oversight of prehospital transfusion training programs.

All seven Canadian CCTOs (100%) used structured training programs incorporating online modules, in-class lectures, practical workshops, simulation, and field training under mentorship (Table 1). Six CCTOs (86%) reported using a combination of these methods, while one CCTO (14%) relied solely on field mentorship. Simulation-based education was widely implemented, with six CCTOs (86%) integrating high-fidelity mannequins, part-task trainers, and screen-based educational modules into training programs. Other technology-enhanced simulation methods such as augmented reality, virtual reality, and mixed reality were not reported as being used by CCTOs. No CCTOs reported using cadaver or animal models in their training programs.

**Table 1:** Training methods and simulation usage in prehospital transfusion training programs

Training Methods	Number of Organizations (*n* = 7)	Percentage (%)
Online modules	7	100
In-class lectures	7	100
Practical workshops	7	100
Simulation	7	100
Field mentorship	7	100
Combination of training methods	6	86
High-fidelity mannequins	6	86
Part-task trainers	6	86
Screen-based educational modules	6	86
Technology-enhanced*	0	0
Cadaver models	0	0
Animal models	0	0

*Technology-enhanced simulation includes augmented reality, virtual reality, and mixed-reality platforms

Instructional design features varied across CCTOs illustrated in Table 2. All seven (100%) CCTOs reported utilizing expert instructor presence. Individualized learning strategies were incorporated into six CCTOs (86%), including features such as self-directed learning, adaptive feedback tailored to learner performance, and opportunities for repeated practice to achieve proficiency, consistent with principles of individualized and mastery-based instructional design. Cognitive task analysis was employed by four CCTOs (57%), while mastery learning and distributed practice were implemented in three CCTOs (43%). Clinical variation, group versus independent practice, and multiple learning strategies were reported by six CCTOs (86%). Repetitive practice was included in five CCTOs (71%), and varying task difficulty was noted in five CCTOs (71%). Feedback mechanisms were used in all seven CCTOs (100%).

**Table 2:** Instructional design features in prehospital transfusion training programs

Instructional Design Features	Number of Organizations (*n* = 7)	Percentage (%)
Cognitive task analysis	4	57
Curricular integration	5	71
Distributed practice	1	14
Expert instructor	6	86
Feedback	7	100
Group practice	6	86
Individualized learning	6	86
Multiple learning strategies	6	86
Range of task difficulty	1	14
Repetitive practice	6	86
Simulation	6	86
Time spent learning	6	86

The use of simulated blood products was examined across all seven CCTOs (100%). Red Blood Cells (RBCs) and FFP were used in training programs by all CCTOs (100%). Platelets were incorporated into training by two CCTOs (29%), while other complex blood products, including Octaplex and fibrinogen, were used by two CCTOs (29%). One CCTO (14%) reported initiating training with whole blood as part of a clinical trial.

CCTOs employed various outcome-measuring tools for competency evaluation. Checklists and global rating scales were used by all seven CCTOs (100%). Time-based assessments, multiple-choice knowledge tests, and participant feedback were reported by five CCTOs (71%). Hand-motion analysis, visuospatial and psychomotor ability testing, and cumulative sum analysis were used by three CCTOs (43%). No CCTOs reported using automated tracking systems for real-time evaluation of training effectiveness.

Competency maintenance and recertification requirements varied across CCTOs. Only one CCTO (14%) required a minimum of three prehospital transfusion initiations per year for providers to maintain competency. The number of required IV initiations per year for competency maintenance was not standardized, with no minimum number reported across all seven CCTOs (100%). Recertification intervals varied, with one CCTO (14%) requiring annual recertification, three CCTOs (43%) requiring bi-annual recertification, and three CCTOs (43%) not implementing a formal recertification process.

Monitoring of transfusion-related performance was inconsistently applied across organizations. Protocol adherence tracking was reported by three CCTOs (43%). Procedural success rate tracking was reported by three CCTOs (43%). Transfusion-related adverse event monitoring was also conducted by three CCTOs (43%). The remaining CCTOs did not report formal tracking mechanisms for transfusion-related performance.

The effectiveness of training programs was evaluated using the Kirkpatrick Model (Table 3). All seven CCTOs (100%) assessed Level 1 (Reaction), where participant engagement and satisfaction with training were measured. All seven CCTOs (100%) evaluated Level 2 (Learning), focusing on knowledge acquisition and skill development. At Level 3 (Behaviour), which examines the application of transfusion skills in clinical practice, five CCTOs (71%) conducted structured evaluations. At Level 4 (Results), which assesses whether training programs impact patient outcomes, no CCTOs reported tracking patient outcomes related to transfusion interventions.

**Table 3:** Kirkpatrick model assessment of prehospital transfusion training programs

Kirkpatrick Model Level	Number of Organizations (*n* = 7)	Percentage (%)
Level 1: Reaction	7	100
Level 2: Learning	7	100
Level 3: Behaviour	4	57
Level 4: Results	0	0

## Discussion

This study identified considerable variability in the structure and delivery of prehospital transfusion training across Canadian CCTOs. Although most organizations reported using a combination of didactic instruction and hands-on modalities, the instructional design features supporting these approaches were inconsistent. When evaluated using the Kirkpatrick Model, training programs primarily emphasized knowledge acquisition (Level 2); however, further research is needed to assess how these efforts translate into clinical practice improvements. Additionally, there was limited implementation of structured evaluations of clinical skill application (Level 3) and no systematic efforts to measure the impact of training on patient outcomes (Level 4). Four CCTOs reported using structured assessment tools to evaluate clinical performance, while none reported routine tracking of transfusion-related outcomes. This gap limits the ability to assess whether training translates into improved patient care.

The absence of standardized competency benchmarks and validated assessment tools likely contributes to differences in skill retention and adherence to best practices across organizations. It is also important to note that many providers in Canadian CCTOs maintain concurrent in-hospital roles, which may support their procedural skill retention outside the prehospital setting. This issue is further compounded by inconsistencies in recertification processes. While some CCTOs required periodic renewal of transfusion training, others had no formal recertification requirement. Without clearly defined evaluation criteria and ongoing skill maintenance strategies, provider proficiency may vary, which could affect the quality and safety of prehospital transfusion care.

Although crew composition was not a specific focus of this study, the results align with previous findings describing variation in staffing models among Canadian CCTOs [6,8]. Depending on the provincial context, teams may be composed of paramedics, registered nurses, or mixed crews [6,8]. Our findings suggest that nurse-led teams more often relied on prior transfusion experience and less frequently employed structured competency assessments. In contrast, paramedic-led teams appeared more likely to adopt formal training frameworks, including simulation-based validation and structured learning pathways. These patterns may reflect broader differences in professional education and clinical governance models. Further research is warranted to understand how crew composition influences training needs, competency assurance, and protocol adherence in prehospital transfusion.

The inconsistent use of simulation-based education also underscores the need for greater standardization. Although high-fidelity simulation is widely supported in the literature for its ability to improve procedural competency, situational awareness, and crisis management, not all CCTOs incorporated it into transfusion training [26,28]. Similarly, the application of evidence-based instructional design principles such as cognitive task analysis, distributed practice, and mastery learning remained limited. These approaches have demonstrated effectiveness in improving clinical performance and skill retention in other domains and could provide important benefits if more broadly adopted within prehospital transfusion training programs [21,22,31].

Given the limited literature on prehospital transfusion training, direct comparisons to previous studies are challenging. However, the growing evidence supporting prehospital blood administration indicate a growing recognition of its role as a life-saving intervention [32,33]. Despite this, training approaches vary widely across jurisdictions, with no universally accepted framework guiding competency development. While structured transfusion training programs integrated with hospital-based transfusion services have been described, evidence on their implementation and effectiveness in prehospital environments remains scarce [34,35]. In Canada, the absence of standardized national guidelines has led each CCTO to develop its own training structure, resulting in inconsistencies in competency benchmarks and transfusion protocols [7,24]. Although some international initiatives have emphasized the need for transfusion training in military and austere settings, there is no standardized approach for civilian CCTOs [36]. This variability underscores the need for further research and the development of a standardized national competency framework to ensure consistent training, provider preparedness, and optimal patient outcomes.

This study has several limitations. The small sample size, despite achieving participation from seven provincial CCTOs, may limit the generalizability of findings beyond the Canadian context. Additionally, variations in Emergency Medical Services (EMS) system design, provider roles, and transfusion protocols across jurisdictions, especially outside Canada, may limit the applicability of these findings in international or non-transport settings. The use of a survey-based methodology introduces potential recall bias, as responses rely on self-reporting rather than direct observational data. The absence of objective performance assessments prevents a comprehensive evaluation of skill acquisition, competency maintenance, and adherence to best practices. Given the complexity of some survey items there is a possibility that respondents may have interpreted some options differently in the absence of interviewer clarification. While this study assessed training structures and instructional design features, it did not directly measure training impact on provider decision-making or transfusion-related patient outcomes. Future studies incorporating direct observation, skill validation, and patient outcome data are necessary to assess the effectiveness of different training approaches in prehospital transfusion care. Moreover, as a descriptive survey study, our findings do not establish a causal link between specific training practices and improvements in clinical performance or patient outcomes.

The variability in transfusion training among Canadian CCTOs highlights the need for nationally standardized guidelines to ensure consistent provider competency and optimal patient care. Standardized competency assessments, simulation-based training, and structured recertification may support skill retention, enhance decision-making, and promote adherence to evidence-based transfusion protocols, though this was not directly evaluated in our study. Future research should focus on establishing a national framework for prehospital transfusion training, integrating simulation-based education into curricula, and implementing structured competency assessments to ensure proficiency across all providers. Additionally, the development of a national prehospital transfusion registry could facilitate the collection of transfusion-related performance data, allowing organizations to track provider competency, adherence to protocols, and patient outcomes. By prioritizing standardized training and competency assessment, Canadian CCTOs can enhance the quality and safety of prehospital transfusion care nationwide.

## Conclusion

Considerable variability exists in prehospital transfusion training across Canadian CCTOs, particularly regarding competency assessment, certification renewal, and patient outcome monitoring. Establishing national training standards with simulation integration and formalized evaluations may support improved provider preparedness and contribute to enhanced patient care, although further evaluation is needed.

## Electronic supplementary material

Below is the link to the electronic supplementary material.

Supplementary Material 1

## Data Availability

Access to the research data would require both REB approval and a data-sharing agreement.

## Abbreviations

AR: Augmented Reality
BCEHS: British Columbia Emergency Health Services
CAN-PATT: Canadian Prehospital and Transport Transfusion
CCTO: Critical Care Transport Organization
CTA: Cognitive Task Analysis
CROSS: Consensus-based Checklist for Reporting of Survey Studies
DLN: Defence Learning Network
EMS: Emergency Medical Services
EVAQ: Évacuations Aéromédicales du Québec
FFP: Fresh Frozen Plasma
IO: Intraosseous
IV: Intravenous
MR: Mixed Reality
RBC: Red Blood Cells
REB: Research Ethics Board
STARS: Shock Trauma Air Rescue Service
VR: Virtual Reality
