# Supplementary material for: Prehospital transfusion training in Canada: a national survey of critical care transport organizations
Source: Scand J Trauma Resusc Emerg Med. 2025 Jul 1;33:114. doi: 10.1186/s13049-025-01435-x (PMC12211276; doi:10.1186/s13049-025-01435-x)
Supplement: Supplementary file 1 — Supplementary Material 1 [file 13049_2025_1435_MOESM1_ESM.docx]

**Prehospital transfusion training in Canada: a national survey of critical care transport organizations**

**Supplementary Materials**

Appendix 1. Checklist for survey: CROSS. 2-4

Appendix 2. Survey. 5-13

Appendix 1. Checklist for survey: CROSS.

| Section/topic | Item | Item description | Reported on page # |
| --- | --- | --- | --- |
| Title and abstract | | |  |
| Title and abstract | 1a | State the word “survey” along with a commonly used term in title or abstract to introduce the study’s design. | Abstract |
|  | 1b | Provide an informative summary in the abstract, covering background, objectives, methods, findings/results, interpretation/discussion, and conclusions. | Abstract |
| Introduction | | |  |
| Background | 2 | Provide a background about the rationale of study, what has been previously done, and why this survey is needed. | 1-2 |
| Purpose/aim | 3 | Identify specific purposes, aims, goals, or objectives of the study. | 2 |
| Methods | | |  |
| Study design | 4 | Specify the study design in the methods section with a commonly used term (e.g., cross-sectional or longitudinal). | 3 |
|  | 5a | Describe the questionnaire (e.g., number of sections, number of questions, number and names of instruments used). | 4 |
| Data collection methods | 5b | Describe all questionnaire instruments that were used in the survey to measure particular concepts. Report target population, reported validity and reliability information, scoring/classification procedure, and reference links (if any). | 4 |
|  | 5c | Provide information on pretesting of the questionnaire, if performed (in the article or in an online supplement). Report the method of pretesting, number of times questionnaire was pre-tested, number and demographics of participants used for pretesting, and the level of similarity of demographics between pre-testing participants and sample population. | 4 |
|  | 5d | Questionnaire if possible, should be fully provided (in the article, or as appendices or as an online supplement). | Appendix 2 |
| Sample characteristics | 6a | Describe the study population (i.e., background, locations, eligibility criteria for participant inclusion in survey, exclusion criteria). | 3-4 |
|  | 6b | Describe the sampling techniques used (e.g., single stage or multistage sampling, simple random sampling, stratified sampling, cluster sampling, convenience sampling). Specify the locations of sample participants whenever clustered sampling was applied. | 4 |
|  | 6c | Provide information on sample size, along with details of sample size calculation. | N/A |
|  | 6d | Describe how representative the sample is of the study population (or target population if possible), particularly for population-based surveys. | 4 |
| Survey  administration | 7a | Provide information on modes of questionnaire administration, including the type and number of contacts, the location where the survey was conducted (e.g., outpatient room or by use of online tools, such as SurveyMonkey). | 4 |
|  | 7b | Provide information of survey’s time frame, such as periods of recruitment, exposure, and follow-up days. | 3 |
|  | 7c | Provide information on the entry process:  –>For non-web-based surveys, provide approaches to minimize human error in data entry.  –>For web-based surveys, provide approaches to prevent “multiple participation” of participants. | N/A |
| Study preparation | 8 | Describe any preparation process before conducting the survey (e.g., interviewers’ training process, advertising the survey). | 4 |
| Ethical considerations | 9a | Provide information on ethical approval for the survey if obtained, including informed consent, institutional review board [IRB] approval, Helsinki declaration, and good clinical practice [GCP] declaration (as appropriate). | 10 |
|  | 9b | Provide information about survey anonymity and confidentiality and describe what mechanisms were used to protect unauthorized access. | 4 |
| Statistical  analysis | 10a | Describe statistical methods and analytical approach. Report the statistical software that was used for data analysis. | 7 |
|  | 10b | Report any modification of variables used in the analysis, along with reference (if available). | N/A |
|  | 10c | Report details about how missing data was handled. Include rate of missing items, missing data mechanism (i.e., missing completely at random [MCAR], missing at random [MAR] or missing not at random [MNAR]) and methods used to deal with missing data (e.g., multiple imputation). | 7 |
|  | 10d | State how non-response error was addressed. | N/A |
|  | 10e | For longitudinal surveys, state how loss to follow-up was addressed. | N/A |
|  | 10f | Indicate whether any methods such as weighting of items or propensity scores have been used to adjust for non-representativeness of the sample. | N/A |
|  | 10g | Describe any sensitivity analysis conducted. | N/A |
| Results | | |  |
| Respondent characteristics | 11a | Report numbers of individuals at each stage of the study. Consider using a flow diagram, if possible. | 8 |
|  | 11b | Provide reasons for non-participation at each stage, if possible. | N/A |
|  | 11c | Report response rate, present the definition of response rate or the formula used to calculate response rate. | 8 |
|  | 11d | Provide information to define how unique visitors are determined. Report number of unique visitors along with relevant proportions (e.g., view proportion, participation proportion, completion proportion). | N/A |
| Descriptive  results | 12 | Provide characteristics of study participants, as well as information on potential confounders and assessed outcomes. | 8-11 |
| Main findings | 13a | Give unadjusted estimates and, if applicable, confounder-adjusted estimates along with 95% confidence intervals and p-values. | N/A |
|  | 13b | For multivariable analysis, provide information on the model building process, model fit statistics, and model assumptions (as appropriate). | N/A |
|  | 13c | Provide details about any sensitivity analysis performed. If there are considerable amount of missing data, report sensitivity analyses comparing the results of complete cases with that of the imputed dataset (if possible). | N/A |
| Discussion | | |  |
| Limitations | 14 | Discuss the limitations of the study, considering sources of potential biases and imprecisions, such as non-representativeness of sample, study design, important uncontrolled confounders. | 12-13 |
| Interpretations | 15 | Give a cautious overall interpretation of results, based on potential biases and imprecisions and suggest areas for future research. | 13-14 |
| Generalizability | 16 | Discuss the external validity of the results. | 14 |
| Other sections | | |  |
| Role of funding source | 17 | State whether any funding organization has had any roles in the survey’s design, implementation, and analysis. | 15 |
| Conflict of interest | 18 | Declare any potential conflict of interest. | 15 |
| Acknowledgements | 19 | Provide names of organizations/persons that are acknowledged along with their contribution to the research. | 15 |
|  |  |  |  |

**Appendix 2. Survey.**

Section 1: Eligibility

1. Are you a healthcare provider, educator, or leader in a Canadian EMS, military, critical care transport or retrieval organization?

Yes

No

Section 2: Demographic Information

2. Which critical care transport organization (CCTO) are you currently affiliated with? (Choose one or specify under 'Other')

British Columbia Emergency Health Services (BCEHS)

Shock Trauma Air Rescue Service (STARS) Alberta

Shock Trauma Air Rescue Service (STARS) Saskatchewan

Shock Trauma Air Rescue Service (STARS) Manitoba

Ornge (Ontario)

Emergency Health Services LifeFlight (Nova Scotia)

Évacuations aéromédicales du Québec (EVAQ)

Canadian Armed Forces

Other (Please specify)

3. What is your current level of care in prehospital services? (Choose one or specify under 'Other')

Emergency Medical Technician (EMT)

Primary Care Paramedic (PCP)

Advanced Care Paramedic (ACP)

Critical Care Paramedic (CCP)

Registered Nurse (RN)

Respiratory Therapist (RRT)

Medical Assistant (Med A)

Medical Technician (Med Tech)

Special Operations Medical Technician (SOMT)

Physician Assistant (PA)

Medical Doctor (MD)

Other (Please specify)

4. What is your current role in prehospital services? (Choose one or specify under 'Other')

Frontline Provider/Clinician

Educator

Logistics

Management and Leadership

Researcher

Other (Please specify)

5. What is your highest level of education achieved? (Choose one or specify under 'Other')

Certificate

College Diploma

Bachelor's Degree

Graduate Diploma or Certificate

Master's Degree

Doctorate (PhD)

Medical Doctor (MD)

Other (Please specify)

6. How many years of experience do you have in the prehospital field?

Less than 1 year

1 to 5 years

6 to 10 years

11 to 20 years

Over 20 years

7. What is your primary work environment? (Select all that apply or specify under 'Other')

Remote

Rural

Suburban

Urban

Metropolitan

Military/Tactical

Mixed Environments (Please define)

Other (Please specify)

8. What is your primary conveyance method? (Select all that apply or specify under 'Other')

Fixed Wing

Rotor Wing

Land Ambulance

Bicycle

Foot Patrol

Mixed

Other (Please specify)

9. What is your primary location of service in Canada? (Select your location)

Alberta

British Columbia

Manitoba

New Brunswick

Newfoundland and Labrador

Northwest Territories

Nova Scotia

Nunavut

Ontario

Prince Edward Island

Quebec

Saskatchewan

Yukon

Other (Please specify)

Section 3: Roles and Training in Prehospital Transfusion

10. What is your role or involvement with prehospital transfusion? (Select all that apply or specify under 'Other')

Administering transfusions

Supervising or managing transfusion procedures

Training personnel in transfusion protocols

Developing transfusion protocols

Conducting transfusion-related research

Policy development

No direct involvement, but interested

Other (Please specify)

11. What is the total duration of your involvement in prehospital transfusion?

Less than 6 months

6 months to 1 year

1 to 5 years

5 to 10 years

Over 10 years

Not applicable

12. What type of prehospital transfusion training have you completed? (Select all that apply or specify under 'Other')

Bloody Easy for Healthcare Professionals

Bloody Easy Blood Administration (BEBA)

Bloody Easy Lite

Safe Transfusion Practice Course

University of Toronto Transfusion Boot Camp

Did not complete transfusion training

Other (Please specify)

13. Which prehospital transfusion training methods have you previously experienced? (Select all that apply or choose 'Not applicable')

Online modules

In-class lectures

Practical workshops

Simulations

Field training under mentorship

Peer-to-peer training

Not applicable

Other (Please specify)

14. What is the duration of the prehospital transfusion training program you have completed?

Less than a full day

Full day

1-2 days

3-7 days

More than 1 week

Other (Please specify)

15. What is the frequency of renewing or recertifying your prehospital transfusion training?

Monthly

Quarterly

Semi-annually

Annually

Other (Please specify)

16. Do you have any formal training in ultrasound-guided IV insertion?

Yes

No

17. How often do you use ultrasound to guide or assist in difficult peripheral IV access? (Please indicate the frequency of use)

Never

Rarely

Sometimes

Often

Always

18. What challenges have you faced during your prehospital transfusion training and practice? (Briefly describe any encountered challenges, such as patient conditions, equipment issues, etc.)

19. What are your preferred methods for prehospital transfusion training? (Rank in order of preference)

|  | 1 | 2 | 3 | 4 | 5 |
| --- | --- | --- | --- | --- | --- |
| Online modules |  |  |  |  |  |
| In-class lectures |  |  |  |  |  |
| Practical workshops |  |  |  |  |  |
| Simulations |  |  |  |  |  |
| Field training under mentorship |  |  |  |  |  |

20. Are you interested in additional prehospital transfusion-related training?

Very interested

Interested

Neutral

Not interested

Not at all interested

21. Are you involved with any regulatory or training bodies related to prehospital transfusion?

Yes

No

If yes, please complete section 4. If no, continue to section 5 (page 11).

Section 4: Education and Competencies in Prehospital Transfusion

22. Which methods of prehospital transfusion training does your organization currently use? (Select all that apply or choose 'Not applicable')

Online modules

In-class lectures

Practical workshops

Simulations

Field training under mentorship

Not applicable

Other (Please specify)

23. Which technology-enhanced simulation methods are currently utilized by your organization for prehospital transfusion training? (Select all that apply or choose 'Not applicable')

Part-task trainers

Cadaver models

Animal models

Augmented reality

Virtual reality

Mixed reality

Screen-based educational teaching modules

High-fidelity Mannequins

No formal simulation training

Other (Please specify)

24. Which instructional design features are currently utilized by your organization for prehospital transfusion training? (Select all that apply or choose 'Not applicable')

Simulator type

Cognitive task analysis

Expert instructor presence

Clinical variation

Curricular integration

Distributed practice (involving >1 day of simulation training)

Feedback

Group (vs independent) practice (>2 learners)

Individualized learning

Mastery learning

Multiple learning strategies

Range of task difficulty

Repetitive practice

Time spent learning

Technology-augmented

Not applicable

Other (Please specify)

25. Which type of simulated blood products are currently utilized by your organization for prehospital transfusion training? (Select all that apply or choose 'Not applicable')

Red Blood Cells (RBCs)

Fresh Frozen Plasma (FFP)

Freeze-dried Plasma (FDP)

Platelets (PLT)

Whole Blood (WB)

Not applicable

Other (Please specify)

26. Which outcome-measuring tools are currently utilized by your organization for prehospital transfusion training? (Select all that apply or choose 'Not applicable')

Time

Multiple-choice questions

Hand-motion analysis

Visuospatial and psychomotor ability

Checklist

Global rating scale (GRS)

Combined checklist and GRS

Cumulative sum analysis

Success rate

Participant feedback

Not applicable

Other (Please specify)

27. In your organization, what is the minimum number of prehospital transfusion initiations for providers to maintain their currency/competence? (Specify number per time period)

28. In your organization, what is the minimum number of successful IV/IO initiations for prehospital providers to maintain their currency/competence? (Specify number per time period)

29. In your organization, what is the frequency of prehospital transfusion training renewal or recertification for providers to maintain their currency/competence?

Monthly

Quarterly

Semi-annually

Annually

Bi-annual

Other

30. Additional information about your organization’s prehospital transfusion training program: (Please provide any information regarding your organization’s prehospital transfusion training program outline, objectives, and relevant details)

Section 5: Feedback and Additional Comments

31. Additional insights: (Please provide comments or suggestions regarding prehospital transfusion practices, training, etc.)
